# A prospective cohort study of the effectiveness of the primary hospital management of all snakebites in Kurunegala district of Sri Lanka

**DOI:** 10.1371/journal.pntd.0005847

**Published:** 2017-08-21

**Authors:** Seyed Shahmy, Senanayake A. M. Kularatne, Shantha S. Rathnayake, Andrew H. Dawson

**Affiliations:** 1 South Asian Clinical Toxicology Research Collaboration, Faculty of Medicine, University of Peradeniya, Peradeniya, Sri Lanka; 2 Department of Medicine, Faculty of Medicine, University of Peradeniya, Peradeniya, Sri Lanka; 3 Central Clinical School, University of Sydney, Sydney, Australia; Universidad Peruana Cayetano Heredia, PERU

## Abstract

**Introduction:**

Sri Lanka records substantial numbers of snakebite annually. Primary rural hospitals are important contributors to health care. Health care planning requires a more detailed understanding of snakebite within this part of the health system. This study reports the management and epidemiology of all hospitalised snakebite in the Kurunegala district in Sri Lanka.

**Methodology:**

The district has 44 peripheral/primary hospitals and a tertiary care hospital-Teaching Hospital, Kurunegala (THK). This prospective study was conducted over one year. All hospitals received copies of the current national guidelines on snakebite management. Clinical and demographic details of all snakebite admissions to primary hospitals were recorded by field researchers and validated by comparing with scanned copies of the medical record. Management including hospital transfers was independently assessed against the national guidelines recommendation. Population rates were calculated and compared with estimates derived from recent community based surveys.

**Results:**

There were 2186 admissions of snakebites and no deaths in primary hospitals. An additional 401 patients from the district were admitted directly to the teaching hospital, 2 deaths were recorded in this group. The population incidence of hospitalized snakebite was 158/100,000 which was significantly lower than community survey estimates of 499/100,000. However there was no significant difference between the incidence of envenomation of 126/100,000 in hospitalised patients and 184/100,000 in the community survey. The utilisation of antivenom was appropriate and consistent with guidelines. Seventy patients received antivenom. Anaphylactic reactions to antivenom occurred in 22 patients, treatment reactions was considered to be outside the guidelines in 5 patients. Transfers from the primary hospital occurred in 399(18%) patients but the majority (341) did not meet the guideline criteria. A snake was identified in 978 cases; venomous snakebites included 823 hump-nosed viper (Hypnalespp), 61 Russell’s viper, 14 cobra, 13 common krait, 03 saw scaled viper.

**Conclusions:**

Primary hospitals received a significant number of snakebites that would be missed in surveys conducted in tertiary hospitals. Adherence to guidelines was good for the use of antivenom but not for hospital transfer or treatment of anaphylaxis. The large difference in snakebite incidence between community and hospital studies could possibly be due to non-envenomed patients not presenting. As the majority of snakebite management occurs in primary hospitals education and clinical support should be focused on that part of the health system.

## Author summary

Snakebite is a neglected tropical disease which mainly affects the rural population in tropical countries. In Sri Lanka the disease burden is an important cause of hospital admission (> 40,000 recorded annually). Most of the previous epidemiological studies in the Island have been done in tertiary care hospitals. We studied the snakebite admissions in all inpatient health facilities (44 primary hospitals and a tertiary care teaching hospital) in an entire rural district where the one of the highest incidence of snakebites reported. The Peripheral hospitals received the majority of snakebites that would be missed in the previous surveys conducted in tertiary care Hospitals. Such an approach is likely to incorporate significant referral bias which may not provide accurate data for health planning. Antivenom is freely available to Sri Lankan government hospitals. All peripheral hospitals keep antivenom and national guidelines are available on management of snakebites. Our data shows that the majority of snakebites in the district are successfully cared for within the primary hospitals only a few needing antivenom therapy. Most of the transfers were not indicated when compared to the criteria described in the national guidelines. Further education and confidence building in management of snakebite is recommended among all categories of health staff in the primary hospitals.

## Introduction

Snakebite is a neglected health issue, mostly affecting rural agricultural communities of the developing countries [1].Over 40,000 hospital admissions due to snakebites are officially recorded in Sri Lanka [2]. Hump-nosed pit vipers (*Hypnale* spp.), Russell's viper (*Daboia russelii*), common krait (Bungarus caeruleus), cobra (Naja naja), saw-scaled vipers (*Echis carinatus*) and several mildly venomous and non-venomous snake species are responsible for snakebites in Sri Lanka. Of these, Russell's vipers, common krait, cobra, saw-scaled vipers and the Hypnale species are the medically important snakes[3].

Sri Lanka is divided into three climatic zones based on rainfall: wet, dry and intermediate [4],[5].The differences in rainfall have led to much diversity in the flora and fauna, and in land use in these zones, leading to differences in snakebite patterns [6]. Its dry zone reports the highest incidence of snakebite resulting high morbidity mortality and economic hardships.

Sri Lanka has a well developed network of government hospitals which provide treatment at no cost to the patients. Within any district there are a number of small primary hospitals supported by at least one referral hospital. It is estimated that no Sri Lankan is more than 30 minutes from a primary hospital. When indicated the government hospital system can offer advanced supportive care (ventilation, dialysis) and imported Indian Polyvalent Antivenom. There are established national guidelines on the management of snakebite produced and distributed by the Sri Lankan Medical Association [7]. These guidelines provide advice on management of envenomation from both identified and unidentified snakes, treatment of anaphylactic adverse reactions to antivenom and indications for interhospital patient transfer.

The actual burden of snakebite for primary hospitals has not been described. While this can be inferred from community surveys of snakebites such surveys have not been validated in predicting hospitalization for snakebite[8]. Previous studies of hospitalized snakebites have been limited to tertiary care hospitals which are potentially biased by referral patterns. This study tests the validity of community surveys as a method to predict hospital admission, quantifies the burden of snakebite in the primary hospital system and measures care delivered against the benchmark of the national guidelines within an entire predominately rural district.

## Methods and materials

### Study designs and patient enrolment

This observational prospective study was conducted in all of the inpatient health facilities in Kurunegala district of North Western Province (NWP) of Sri Lanka. The province is bound to dry and wet climate zones where the one of the highest incidence of snake bites reported.

All patients who presented with snakebite between the 25th May 2013 and 25^th^ May 2014 to any of the 44 primary hospitals or to the tertiary Teaching Hospital Kurunegala (THK) in Kurunegala district were enrolled in the cohort. The Primary Hospitals includes the Base Hospitals and the Divisional Hospitals. The Base Hospitals provide health care in relation to four main specialties only. The Divisional Hospitals are hospitals with very low facilities and they do not provide any specialized care. Data linkage was undertaken to identify the outcome of any patient transferred from a primary hospital to the THK. After date linkage for transfers all subsequent analysis was anonymized.

The 2012 Sri Lankan census was used for estimating district population[9,10].

A previously published community survey of snakebite within the district was undertaken between August 2012 to June 2013 was used to compare with the population incidence of hospitalized snakebite calculated in this study [8].

### Procedures

The data extracted from the patients’ hospital records were transcribed into data extraction forms, and entered into a database in Microsoft Access by trained research assistants. The main variables extracted were demographic information, identification of snakes, clinical signs and symptoms treatment. A scanned copy of hospital record was attached to the data record.

### Snake identification, clinical assessment and treatments

Snakes were identified by hospital staff if the snake specimen was brought to the hospital using the SLMA guidelines for the identification of snakes [7]. In the absence of the snake being brought to hospital snake were identified from history of patients and witnesses. The major determinant for use of polyvalent antivenom was the clinical syndrome of envenomation[7]. Syndromic diagnosis has been demonstrated to have a high specificity for species diagnosis [11].This is important in identifying Hypnale hypnale envenomation as polyvalent antivenom is ineffective and not indicated. Recorded data included epidemiological and demographic details, clinical features of envenoming such local pain, swelling, necrosis and coagulopathy, neurotoxicity and other systemic symptoms. Details of antivenom administration, premedication against adverse drug reactions and management of adverse reactions were recorded.

### Expert review

Using predefined criteria based on SLMA guideline[7] an expert clinician independently reviewed and scored scanned copies of the hospitals record to identify; the appropriateness of transfers to tertiary care centers, accuracy of identification of offending snakes, evidence of envenoming, indication for antivenom (AV) therapy, regimen and dose of AV therapy, management of AV adverse reactions and appropriateness of overall patient management. The accepted indications of transfers were anticipated ventilatory problems, need of ICU care, severe coagulopathy, impending acute kidney injury, need of surgical care for local necrosis and refractory shock, lack of resuscitation facilities, AV and emergency medications.

### Data management and statistics

Data were entered into a database in Microsoft Access by trained research assistants. The database was independently cross checked with the original data sources for accuracy and completeness by two other researchers. Data analysis was performed in the R programing language version 3.2.5.

All the individual level variables including the signs and symptoms for envenoming and the incidence of snake bites were considered for descriptive analysis.

### Ethics statement

The study was approved by the Ethical Review Committee, Faculty of Medicine, University of Peradeniya, Sri Lanka. As the study was an audit of clinical practice undertaken in collaboration with the treating Health Authority, North Western Province. Provincial Health individual patient consent was not required by the IRB.

## Results

There were 2186 admissions of snakebites reported from the primary rural hospitals. There was an additional 401 patients from the district who presented directly to THK (Table 1). Thus, total snakebite in the district was 2587 with population incidence was 158 (95% CI 134–185) cases per 100,000 population. There were two deaths in the cohort, both patients had been direct admissions to THK. There were no deaths of patients who presented to a primary hospitals or subsequently transferred to THK.

**Table 1:** Distribution of total and direct adult snakebite admission to THK.

Description n (%)	Direct	Total (Direct+ Transfers)
Number	401(54)	737
Type of Bite		
Hump -nosed viper	176(44)	263(36)
Russell’s viper	25(6)	63(8)
Cobra	10(2)	22(3)
Krait	07(2)	14(2)
Cat snake	02(0.5)	04(0.5)
Water snake	01(0.25)	02(0.3)
Wolf snake	03(0.75)	03(0.4)
Rat snake	02(0.5)	02(0.3)
Unidentified snakes	175(44)	364(49)
Total Number of antivenom given	35(9)	95(13)
Deaths	02(0.5)	02(0.3)

*THK-Teaching Hospital Kurunegala

### Demographic and epidemiological data

The median age of the patients presenting to the primary hospital was 40 years (IQR 27–53), and 59% were males. Median time to hospital arrival was 45 minutes (IQR 30–90) and 49% of bites occurred between 6pm and 12am (S1 Table). The offending snake was identified in 978(45%) cases: 03 mildly venomous species, 61 non-venomous snakes and 914 venomous snakes. The venomous snakebites included 823 hump-nosed viper (Hypnale spp), 61 Russell’s viper, 14 cobra, 13 common krait, 03 saw scaled viper and 3 Green pit vipers. Of the identified, 91 cases (9%) the live or death specimen were brought to the study hospitals (S2 Table). Antivenom was given to 70(3%) patients and 22(31%) of were documented to have developed anaphylactic reactions.

### Clinical manifestations

Sign or symptoms of envenoming were detected in 1690 cases (77.3%) in the primary hospitals and 383 (95.5%) direct admissions to THK including the unidentified bites. The total district incidence of envenomed was 126 (95% CI 105–150) cases per 100,000 population.

Of the envenomed cases in the primary hospitals, local envenomation was detected in 1510 cases (89%), non-specific systemic effects such as abdominal pain, nausea, vomiting, diarrhea, chest pain and headache in 259 cases (15.3%) and, the specific systemic effects such as coagulopathy, neurotoxicity, spontaneous bleeding and nephrotoxicity in 359 cases (21%). These clinical manifestations occurred either as sole manifestation or in combination with others (S3 Table).

### Treatment outcome

Of the 2186 cases, antivenom has been given to 70 (3%) patients as they had signs of systemic envenoming. Of them, 22 had developed anaphylactic reactions to antivenom, only 6 were judged to be severe (Table 2). Treatment was indicated in all patients, there was no record of treatment for 3 patients. All other patients received treatment that was appropriate for the severity of the reaction; 73% received adrenaline, 54% hydrocortisone, 28% IV antihistamine (either promethazine or chlorphenamine).

**Table 2:** Description of antivenom (AV) therapy, anaphylaxis reactions and the management of them.

Description	n(%)
Total AV given	70(3.2)
Reactions ^a^ to AV	22(31)
• Mild	3 (14)
• Moderate	13(59)
• Severe	6(27)
Treatment to anaphylaxis	
• IV Hydrocortisone	12(54)
• Adrenaline	16(73)
• IV Chlorphenamine	3(14)
• IV Promethazine	3(14)
• Dopamine	3(14)
• Other(maxolon, Salbutamol, Hartman)	3(14)
• No treatment	3(14)

^a^ In all cases there is itching, Urticaria and rigors. Mild-the Blood Pressure is normal; moderate and severe reactions–there is hypotension and bronchospasm (SLMA guideline)

All the patients with severe anaphylactic reactions had the hypotension with the range of systolic blood pressure from 60 to 70. One patient had unrecordable BP and pulse. Of them, 04 received the repeated doses of adrenaline, 03 treated with dopamine, 05 with IV hydrocortisone, 03 with IV Promethazine and 01 patient nebulized with oxygen.

### Expert opinion and outcome

The indications for the use of antivenom in the primary hospitals was considered appropriate in all cases. In 6% (4/70) of patients the dose of antivenom used was less than that recommended by the national guidelines. No patients who remained in the primary hospital were identified as inappropriately not receiving antivenom when antivenom treatment was indicated.

**Fig 1:**
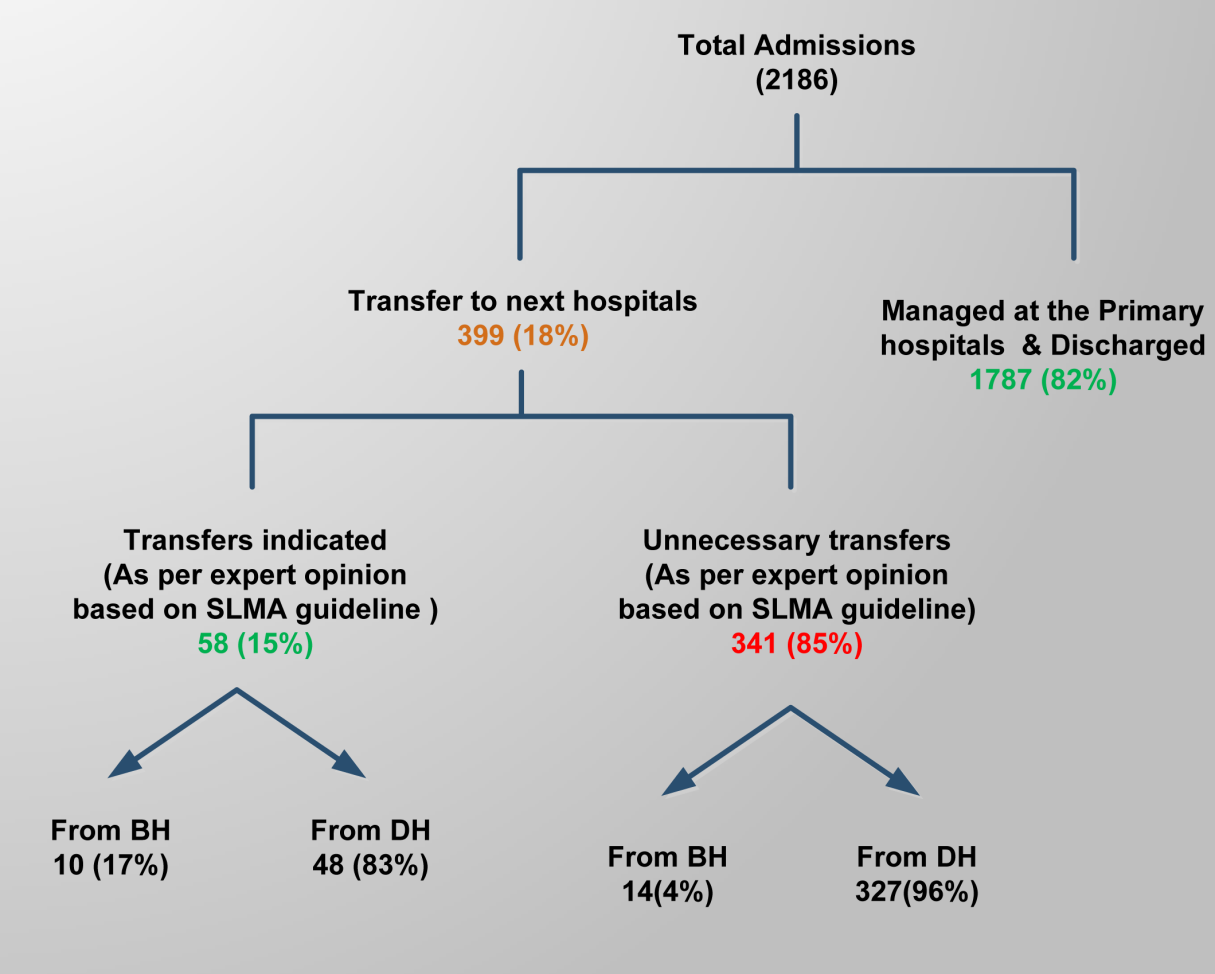
Distribution of transfers from primary hospitals as per predefined expert criteria based on SLMA guideline. BH- Base Hospital, DH-Divisional Hospital; SLMA guidelines-Sri Lanka Medical Association guidelines for the management of snakebite in Hospitals.

Of the total, 399(18%) patients were transferred out from primary hospitals. Only 58 transfers were indicated according to the apriori expert criteria (Fig 1). The most common reason for transfer was systemic envenomation with unpredictable outcome (Table 3). Documentation in the patient notes for the reason for transfer was only found in 84 records with only 19 meeting transfer criteria (S4 Table). Within the transfer group 105 patients were transferred to a facility in another health district and 294 cases were transferred to THK. In the transfer to THK hospital case records of 177 were available for analysis, 30 cases (17%) received AVS at THK 14 of these patients were identified by the expert review as having indications for antivenom in their primary hospital admission record.

**Table 3:** The indications for transfers according to apriori expert criteria (n = 58).

Indication for Transfers (Apriori Expert criteria)	n (%)
Systemic envenoming with unpredictable outcome	37(64)
Pediatric with systemic envenomation	12(21)
Comorbidities	03(5)
Moderate to severe reactions and unpredictable outcome	03(5)
Severe local Necrosis (for surgical management)	03(5)

## Discussion

Previous studies of hospitalised snakebite in Sri Lanka have focused upon tertiary care hospitals [6,12–15]. Such an approach is likely to incorporate significant referral bias which may not provide accurate data for health planning. Our data shows that the majority of snakebites in the district are successfully cared for within the primary hospitals.

Antivenom is freely available to Sri Lankan government hospitals. Lack of antivenom as a cause for transfer was only documented in 2/84 cases. All peripheral hospitals keep antivenom and national guidelines are available on management of snakebites in each hospital [7]. Doctors receive training in snakebite management as undergraduates. Within this district some doctors from each hospital received 1 day of continuing medical education on the use of the national guidelines in mid of 2013.

Antivenom was not indicated for the majority of patients. The median primary hospital stay of patients who did not receive antivenom and were not transferred to another hospital was 37 hrs 29 min (IQR: 20hr 43 min - 47hr 28 min).The low use of antivenom reflects the high proportion (67%) of patients were envenomed by the Hump Nosed Viper for which there is currently no effective antivenom available. These patients still required clinical management of coagulopathy, prevention of acute renal failure with fluid therapy, puncturing of the blisters, prophylactic antibiotic management and elevation of the limb as per SLMA guidelines for managing Hump-Nosed Pit vipers [7].All the hump nosed viper bites were closely monitored for more than 12hrs irrespective to the clinical manifestations (Median 39 hr; IQR- 25 hr 41 min to 53 hr 18 min). It is also likely that some patients with unidentified snakebite due to Hypnale would have received antivenom in the absence of a typical hypnale syndrome. There is significant geographic variation of presentation of hump-nosed viper bite within Sri Lanka. General Hospital, Anuradhapura in the North Central province records about 7% case incidence [12,13]whereas General Hospital Jaffna in the Northern Province records hardly any cases[16]. In comparison, Southern districts of Sri Lanka reports high incidence of hump-nosed viper bites [17–20].

Primary hospitals used antivenom appropriately as indicated by the national guidelines[7]. The reported rate of anaphylactic reactions in this study was 31% (22/70). This is at the low end of the range of 30 to 81% reported from previous RCT antivenom studies and suggests some underreporting or poor documentation [21],[22],[23],[24]. The management of the reactions in primary hospitals was within the SLMA guidelines.

We recorded 399 (18%) transfers to tertiary care hospitals. In our review there was no indication in the clinical record of a need for transfer in 85% of patients (Fig 1). These results were validated in our review of patients after transfer in whom only 17% had an indication for transfer albeit for antivenom. Arguably these patients could have been successfully treated in the primary hospital.

In Sri Lanka inter hospital ambulance costs are borne by the health system and represent a considerable cost relative to a days hospital stay [25]. In those patients who were transferred 30% received antivenom however based upon the data from the cohort the majority of these patients could have been managed in a primary hospital. The primary reason for transfer may include other factors. Previous studies have highlighted that doctors in primary rural hospitals may feel professionally isolated that there is a high level of community influence in treatment decisions involving acutely poisoned patients [26].Despite clear guidelines [7] based on extensive local research [24], [22], [21], [27], [6], [23] rural doctors may be concerned about their ability to treat anaphylactic reactions.

Taking direct admissions to THK into account, the Kurunegala district recorded 2587 cases of snakebite per year. The rate of hospitalized snakebite 158 (95% CI 134–185)/100,000 is much lower than the estimates made in community survey within the same district of 499 (392–605)/100,000 [8]. In our study the rate of patients hospitalized with envenoming was 2073 with population incidence of 126/100,000 (95% CI 105–150) is consistent with the community estimates in the district of 184 (118–251)/100,000 [8]. This may reflect health seeking behavior of individuals, for example a victim of a bite from a snake recognized to be non-venomous may not present to hospital but does suggest that patients with envenomation do present to hospital. The median time of 45min to present to hospital reflects the treatment seeking behavior of the people, availability of transportation, road structure and proximity of the hospitals to the victims.

In this study, no data was available about what first aid or herbal medicine patients used before coming to hospital. A previous hospital based study showed only 4% of victims used some forms of indigenous treatments before coming to hospital[13]. It is possible that indigenous treatments may be used more frequently in patients with presumed lower risk exposures who do not present to hospitals.

Hospital medical staff were able to recognize all dry bites and mild envenoming. Attempts were made to identify offending snakes and signs of envenoming. In 44% of cases offending snakes were identified, but only in limited number of cases dead specimens of snakes were available for confirmation. The epidemiology of snake bite in the district was consistent with the known geographic distribution of snakes. Snakes were not identified in more than 50% (1208) of bites. Among them 315 are dry bites and 189 cases had only pain at the site of bite. Probably, all these cases are non-venomous bite, but less than the incidence of non-venomous bites recorded from central hills of the country in the Central Province [28].

### Implications

This study reveals that the majority (82%) of snake bite in the district is not managed in the tertiary referral hospital but is successfully managed in the primary hospital. This has implications for resource utilization and training. Unnecessary transfers could be addressed with additional doctor and community education. However as the majority of snakebite patients remain in the primary hospitals there may be unmet health needs. Previous research in Sri Lanka in envenomed patients showed that 54% met the criteria for depression and 27% post-traumatic stress disorder with 10% of patients ceasing work[29]. Some symptoms were responsive to brief psychological intervention delivered by non-specialist doctors[30]. Currently there are trials on hump nose antivenom, Given the high prevalence of hump-nosed viper in this other rural regions the introduction of an antivenom will have important implications for health planning as the number of patients who could potentially receive antivenom will dramatically increase. This will increase direct treatment costs as well as transfers.

### Limitations

As in all chart review based studies the record of clinical signs and symptoms is likely to be incomplete. This is obvious with the low rate of recording of explicit reasons for transfer to another hospital. While outcomes of death, transfer and use of antivenom are likely to be robust anaphylactic reactions are most likely underestimates in particular mild ones [27].

Misidentification of offending snakes by hospital staff is a source of error, in a prospective study this occurred in 6% of samples. However clinical syndromes had a high specificity for species identification and should reduce inappropriate antivenom use in H. hypnale [11].Errors in snake identification by patient or bystander is likely to be higher than hospital staff but is not likely to impact on decisions to treat which is based upon clinical envenomation.

## Conclusion

Peripheral hospitals received the majority of snakebites that would be missed in surveys conducted in tertiary hospitals. Snakebites were treated appropriately and effectively in the primary hospitals with only a few needing antivenom therapy. Most of the transfers were unnecessary indicating need of guideline and education on improvement. Further education and confidence building in management of snakebite is recommended among all categories of health staff in the primary hospitals.

## Supporting information

S1 Table General descriptions of the distribution of gender, age, time and site of bite of primary hospital admissions. (DOCX)

S2 Table Identification of snakes (n = 978). (DOCX)

S3 Table Description of clinical features of 2186 snakebite patient. (DOCX)

S4 Table Documented indications for transfers from primary hospitals (n = 84). (DOCX)

S1 Appendix STROBE_checklist.doc. (DOC)
