# Supplementary material for: A prospective cohort study of the effectiveness of the primary hospital management of all snakebites in Kurunegala district of Sri Lanka
Source: PLoS Negl Trop Dis. 2017 Aug 21;11(8):e0005847. doi: 10.1371/journal.pntd.0005847 (PMC5578683; doi:10.1371/journal.pntd.0005847)
Supplement: S1 Table — (DOCX) [file pntd.0005847.s001.docx]

**S1 Table. General descriptions of the distribution of gender, age, time and site of bite of primary hospital admissions.**

| **Description** |  |  |
| --- | --- | --- |
| Gender  (n=2186) | Male | 1289(59%) |
|  | Female | 897(41%) |
|  |  |  |
| Age | Median | 40 |
|  | IQR | 27-53 |
|  |  |  |
| Time of Bite  (n=1399) | 00.00-06.00h | 135(10%) |
|  | 06.00-12.00h | 296(21%) |
|  | 12.00-18.00h | 283(20%) |
|  | 18.00-24.00h | 685(49%) |
|  |  |  |
| Site of bite  (n=1659) | Lower limb | 1265(76%) |
|  | Upper limb | 350(21%) |
|  | Other parts | 44(3%) |
|  |  |  |
