# Supplementary material for: A prospective cohort study of the effectiveness of the primary hospital management of all snakebites in Kurunegala district of Sri Lanka
Source: PLoS Negl Trop Dis. 2017 Aug 21;11(8):e0005847. doi: 10.1371/journal.pntd.0005847 (PMC5578683; doi:10.1371/journal.pntd.0005847)
Supplement: S2 Table — (DOCX) [file pntd.0005847.s002.docx]

**S2 Table. Identification of snakes (n=978)**

| Description  n (%) | HNV^*^  n=823 | RV^*^  n=61 | Cobra  n=14 | Krait  n=13 | SSV^*^  n=3 | GPV^*^  n=3 | Non venomous^*^  n=61 |
| --- | --- | --- | --- | --- | --- | --- | --- |
| Snake seen either by victim or witnesses and examining snake specimen  n=977(99.9) | 822(99.8) | 61(100) | 14(100) | 13(100) | 3(100) | 3(100) | 61(100) |
| Snake brought  to the hospital  n=91(9) | 65(7.9) | 1(1.6) | 1(7.1) | 5(38.5) | 3(100) | 2(66.7) | 14(22.9) |

^*^HNV-Hump-nosed viper, RV-Russell’s viper, SSV-Saw Scaled Viper, GPV-Green pit viper, Non venomous-Cat snake, Rat snake, Python, Water snake, Wolf snake
