# Supplementary material for: A prospective cohort study of the effectiveness of the primary hospital management of all snakebites in Kurunegala district of Sri Lanka
Source: PLoS Negl Trop Dis. 2017 Aug 21;11(8):e0005847. doi: 10.1371/journal.pntd.0005847 (PMC5578683; doi:10.1371/journal.pntd.0005847)
Supplement: S3 Table — (DOCX) [file pntd.0005847.s003.docx]

**S3 Table. Description of clinical features of 2186 snakebite patient.**

| Description  Clinical manifestations | HNV*  n=823 | RV*  n=61 | SSV*  n=3 | GPV*  n=3 | Cobra  n=14 | Krait  n=13 | Non-venomous* (Identified)  n=61 | Unidentified  n=1208 |
| --- | --- | --- | --- | --- | --- | --- | --- | --- |
| n (%)   - Bite Mark 530(24) - Number of envenomed   patients 1690(77)  Local effects  Total =1510(89)   - Pain 1211(80) - Swelling 1045(86) - Blistering/Necrosis45(3) - Cellulites 20(1)   Non Specific  Total=259(15)   - Abdominal Pain95(37) - Chest Pain 26(10) - Nausea 35(13) - Vomiting118(46) - Headache 53(20) - Diarrhoea 2(1)   Systemic envenomed  Total=359(21)   - Hemorrhage 96(27) - Incoagulable blood (20WBCT) 237(66) - Neurological Signs76(21) - Acute Kidney Injury 1 | 210(25)  692(84)  670(81)  517(63)  546(66)  20(2)  11(1)  52(6)  11(1)  4(0.5)  9(1)  22(3)  11(13)  90(11)  29(3.5)  61(7)  3(0.4)^b^ | 17(28)  53(87)  44(72)  40(66)  19(31)  1  16(26)  9(15)  2(3)  10(17)  1  1  25(40)  5(8)  18(30)  13(21) | 3(100)  3(100)  2(67)  1(33)  1(33)  1(33)  1(33)  1(33) | 1(33)  2(67)  2(67)  2(67)  2(67)  1(33)  1(33) | 6(43)  10(71)  6(43)  4(29)  3(21)  1  3(21)  1(7)  2(14)  8(57)  7(50)  2(14) | 2(15)  9(69)  5(38)  4(31)  2(15)  4(31)  2(15)  2(15)  1  4(31)  2(15)  2(15) | 13(21)  28(46)  25(41)  19(31)  7(12)  2  2(3)  1  1  4(7)  4(7) | 281(23)  893(74)  755(62)  623(52)^a^  65(38)  22(2)  8(1)  182(15)  72(6)  20(2)  23  82(7)  40(3)  1  226(19)  58(5)  147(12)  55(5)  1 |

*HNV-Hump-nosed viper, RV-Russell’s viper, SSV-Saw Scaled Viper, GPV-Green pit viper, Non- venomous identified-Cat snake, Rat snake, Python, Water snake, Wolf snake

^a^Only local pain occurred in 189 cases

^b^ Three patients developed neuroparalysis
