# Supplementary material for: A prospective cohort study of the effectiveness of the primary hospital management of all snakebites in Kurunegala district of Sri Lanka
Source: PLoS Negl Trop Dis. 2017 Aug 21;11(8):e0005847. doi: 10.1371/journal.pntd.0005847 (PMC5578683; doi:10.1371/journal.pntd.0005847)
Supplement: S4 Table — (DOCX) [file pntd.0005847.s004.docx]

**S4 Table. Documented Indications for transfers from primary hospitals (n=84).**

| **Indication for transfer** | **n (%)** |
| --- | --- |
| For further management | 30(36) |
| WBCT>20min | 29(34) |
| Reactions to Antivenom/may need ICU care | 06 (7) |
| AKI/suspected AKI/Urine output dropped | 04 (5) |
| For surgical management /Medical Opinion | 03(4) |
| With systemic envenoming/Abdominal pain | 03(4) |
| Pain/Swelling/Rhonchi | 03(4) |
| Bitten by suspected krait/Bitten by HNV/Unknown | 03(4) |
| AVS not available | 02(2) |
| Do not give IM injection (ambiguous statement) | 01(1) |
